# Surface Depassivation via B–O Dative Bonds Affects the Friction Performance of B-Doped Carbon Coatings

**DOI:** 10.1021/acsami.3c18803

**Published:** 2024-03-28

**Authors:** Stefan Peeters, Takuya Kuwahara, Fabian Härtwig, Stefan Makowski, Volker Weihnacht, Andrés Fabián Lasagni, Martin Dienwiebel, Michael Moseler, Gianpietro Moras

**Affiliations:** † Fraunhofer IWM, MiktroTribologie Centrum μTC, Wöhlerstraße 11, 79108 Freiburg, Germany; ‡ Osaka Metropolitan University, 3-3-138 Sugimoto, Sumiyoshi-ku, 558-8585 Osaka, Japan; § Fraunhofer IWS, Winterbergstraße 28, 01277 Dresden, Germany; ∥ Technische Universität Dresden, Institut für Fertigungstechnik, George-Bähr-Straße 3c, 01069 Dresden, Germany; ⊥ Karlsruhe Institute of Technology (KIT), IAM – Institute for Applied Materials, Straße am Forum 7, 76131 Karlsruhe, Germany; # University of Freiburg, Institute of Physics, Herrmann-Herder-Straße 3, 79104 Freiburg, Germany

**Keywords:** diamond-like carbon, boron doping, friction, tribology, dative bond

## Abstract

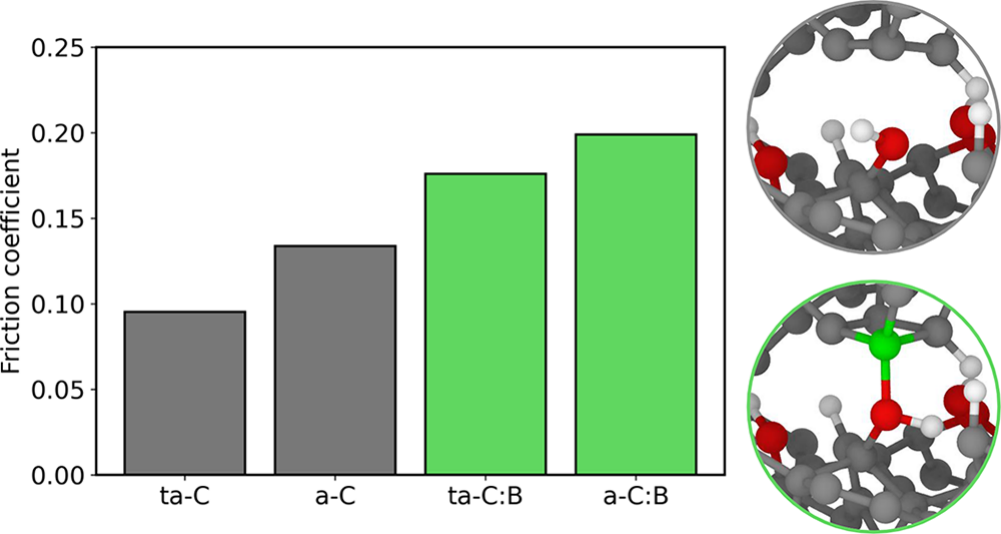

Boron doping of diamond-like carbon coatings has multiple effects on their tribological properties. While boron typically reduces wear in cutting applications, some B-doped coatings show poor tribological performance compared with undoped films. This is the case of the tribological tests presented in this work in which an alumina ball is placed in frictional contact with different undoped and B-doped amorphous carbon coatings in humid air. With B-doped coatings, a higher friction coefficient at a steady state with respect to their undoped counterparts was observed. Estimates of the average contact shear stress based on experimental friction coefficients, surface topographies, and Persson’s contact theory suggest that the increased friction is compatible with the formation of a sparse network of interfacial ether bonds leading to a mild cold-welding friction regime, as documented in the literature. Tight binding and density functional theory simulations were performed to investigate the chemical effect of B-doping on the interfacial properties of the carbon coatings. The results reveal that OH groups that normally passivate carbon surfaces in humid environments can be activated by boron and form B–O dative bonds across the tribological interfaces, leading to a mild cold-welding friction regime. Simulations performed on different tribological pairs suggest that this mechanism could be valid for B-doped carbon surfaces in contact with a variety of materials. In general, this study highlights the impact that subtle modifications in surface and interface chemistry caused by the presence of impurities can have on macroscopic properties, such as friction and wear.

## Introduction

Carbon-based coatings are among the most successful materials to reduce friction and wear in moving parts due to their unique mechanical and chemical properties.^1,2^ Their hardness, thermal conductivity, and chemical inertness make diamond and diamond-like carbon (DLC) coatings particularly effective in bearings and seals,^3,4^ micro- and nanoelectromechanical systems,^5,6^ as well as medical applications.^7^ The tribological performance of carbon-based coatings, however, is influenced by the surface morphology, environmental, pairing,^8−10^ and lubricating conditions.^1,11,12^ Different passivation and friction regimes can be observed depending on relative humidity, surface orientation, and mechanical stresses at play.^12,13^ In the case of self-mated diamond surfaces in dry conditions, interface cold welding and amorphization are observed, resulting in high friction coefficients. In the presence of water, even at low interfacial concentrations, water molecules decompose leading to interfacial ether (C–O–C) bonds, responsible for mild cold-welding, or H/OH/O-passivation, that prevents the formation of interfacial bonds^14,15^ and can enable ultralow friction.^16,17^ These friction regimes are also valid for amorphous carbon surfaces^2^ and for heterogeneous tribological interfaces in which a carbon film is transferred from the carbon coating to the counter surface.^9^

Some industrial applications require coatings with oxidation and wear resistance higher than those typically exhibited by pristine polycrystalline diamond. One possibility to improve the tribological properties and the stability of diamond films in oxidative environments is through boron doping. For instance, B-doped nanopolycrystalline diamond can be used to produce cutting tools able to reach wear rates 3 orders of magnitude lower than the undoped material.^18^ B-doped carbon coatings could also be used in other tribological applications, yet their capability to reduce friction during sliding is still debated. Several authors performed tribological and cutting tests on different carbon coatings and reported a lower friction coefficient and reduced wear on diamond films,^19−23^ as well as hydrogenated amorphous carbon,^24−26^ and enhanced oxidation resistance^27,28^ for the B-doped substrates compared to the undoped ones. In contrast, some studies report no significant improvement^29,30^ or even worse tribological performance,^26,31^ when B is present in the coating. Such a variety of results can be explained by the different preparation procedures of the B-doped diamond and amorphous carbon coatings as well as the different environmental and tribological conditions to which the films were exposed to. Typical grain sizes for the B-doped diamond crystals range from the nano- to the micrometer scale,^20,31^ and improved tribological performance is usually obtained for B concentrations around 0.3% and between 0.2 and 1.0% for diamond^19,22^ and hydrogenated amorphous carbon,^24,25^ respectively. Nevertheless, incomplete experimental characterization of the morphological and mechanical properties of the coatings, different operational conditions, and, in some cases, no estimation of the B or H content in the films make a direct comparison of the results presented in the literature and the understanding of the underlying interfacial phenomena challenging.

Another source of complexity comes from the multiple effects of B-doping on the surface chemistry of the carbon coatings. For example, the enhanced electric conductivity makes B-doped carbon materials particularly valuable for electrochemical and electroanalytical applications.^32^ Graphitization, which is connected to the change in the electronic properties of the carbon material,^33^ was shown to be more likely to occur in the presence of B.^34−36^ This phenomenon can have a strong impact on the tribological properties of the coatings because aromatic structures typically passivate sliding carbon interfaces and provide low friction coefficients.^2,12,13^ Another effect of B that is particularly relevant for tribology is the change in surface reactivity of diamond and diamond-like carbon, as suggested by several computational studies. Latorre et al. demonstrated that the energy barriers associated with the dissociative chemisorption of H_2_, O_2,_ and H_2_O are higher for B-doped diamond than on the undoped surface, yet the dissociation products on the surface of B-doped diamond are more stable than on the undoped surface.^37^ A particularly interesting observation is that while H_2_O adsorption on undoped diamond surfaces occurs either by physisorption or by dissociative chemisorption, a further adsorption configuration of H_2_O is available on the B-doped surface.^37^ The latter is characterized by undissociated chemisorption through a B–O dative bond. Finally, Zhao and Larsson showed that OH groups are more reactive when the C atom which they are attached to is connected to a B atom,^38,39^ while Jaimes et al. reported that the adsorption energy of water and OH groups is more favorable when B is at the diamond surface.^40^

All of these examples raise the question of whether changes in surface chemistry caused by the presence of boron can have a significant influence on the effectiveness of surface passivation of the carbon coatings. Given the considerable importance of stable passivation of surface dangling bonds for the boundary lubrication properties of carbon surfaces, B-induced changes in surface and interface chemistry could in turn significantly affect the tribological performance of B-doped carbon coatings. Motivated by this question, this study starts with ball-on-disc tribological experiments revealing an increase in the coefficient of friction related to the presence of boron in various amorphous carbon coatings. A qualitative estimate of the contact shear stress values based on experimental friction coefficient, surface topographies, and Persson’s contact theory suggests that the increase in friction could be related to the formation of a sparse network of interfacial bonds. A detailed computational study based on tight binding (TB) and density functional theory (DFT) simulations confirms this hypothesis and reveals how B atoms can disable OH surface passivation through the formation of dative B–O bonds, which eventually lead to ether-like bridging bonds across the tribological interface. The results thus show that the effects of doping on the tribological properties of carbon coatings can be caused by subtle changes in surface chemistry and not only more evident modifications of their mechanical properties or microstructure.

## Materials and Methods

### Materials

Polished steel samples (100Cr6, hardened to 60 HRC, *R*_a_ < 0.03 μm, dimension 18 × 13.5 × 3 mm^3^) were coated in a commercial physical vapor deposition (PVD) chamber (VTD Vakuumtechnik Dresden GmbH, Dresden, Germany) with an attached LaserArc carbon evaporation source (Fraunhofer IWS, Dresden, Germany). An 8-axis planetary system was used as a sample holder and set in 2-fold rotation. Standard graphite targets from Plansee Composite Materials GmbH, Germany, were used as the cathode material for the generation of pure carbon coating. In the case of B-doped carbon, powder-pressed and sintered composite graphite targets with a nominal content of 5 at % of boron from the same company were used. The deposition chamber was evacuated to high vacuum at a pressure of about 10^–4^ Pa, and an argon ion etching process was carried out prior to coating deposition. Subsequently, a chromium adhesion interlayer with a thickness of approximately 100 nm was deposited by magnetron sputtering, followed by the deposition of the carbon coatings by pulsed arc discharge that generates the carbon plasma. The arc discharges were ignited by using laser pulses from a Q-switched Nd:YAG laser. A negative bias voltage of 100 V was applied to the substrate holder. Detailed information on the deposition process has been published elsewhere.^41,42^ For the analysis of chemical composition, the boron-doped samples were measured using SEM (acceleration voltage of 10 V, spot size 50, and working distance of 10–13 mm) with an EDS system JEOL 6610 + X-Max 80 mm2 (JEOL, Akishima, Japan and X-MAX 80 from Oxford Instruments plc., Abingdon, U.K.). The chemical composition was determined using AZtec software (version: 3.3). An overview of all the coatings and their properties is given in Table 1, while a complete characterization is available in ref (43).

**Table 1:** Coating Types and Their Properties^a^

coating type	thickness (μm)	hardness (GPa)	Young’s modulus (GPa)	B concentration (%)
**a-C**	1.6–2.1	29.6 ± 1.2	352 ± 18	0.0
**ta-C**	1.9	53.0 ± 0.5	627 ± 13	0.0
**a-C:B**	1.5	18.9 ± 0.4	238 ± 39	5.2
**ta-C:B**	1.4	56.1 ± 2.4	577 ± 12	5.3

a The two coatings with the lowest hardness and Young’s modulus are labeled a-C (amorphous carbon); the other two are labeled ta-C (tetrahedral amorphous carbon).

The coating thickness was measured by using the ball crater method. Hardness and Young’s modulus were determined using a ZHN nanomechanical test system from Zwick/Roell (Ulm, Germany) with the quasi-continuous stiffness method (QCSM). For indentation, a Berkovich tip was used with a maximum load of 100 mN. The coating modulus was calculated as described in EN ISO 14577-4:216, using a sigmoid fit model for extrapolation of the Young’s modulus at the coating surface.^44^ After deposition, all coatings were lapped with a diamond slurry.

The counter bodies for the tribological tests consisted of aluminum oxide (Al_2_O_3_) balls with a diameter of 10 mm and were obtained from Sd Hartstofftechnik (Wuppertal, Germany). This material was selected because of its low reactivity toward carbon-based coatings.

### Tribological Tests

Tribological experiments were performed using a SRV 4 tribometer from Optimol Instruments Prüftechnik GmbH (Munich, Germany) in a reciprocating ball-on-disc configuration. Normal force and sliding duration were determined through preliminary experiments in order to obtain some measurable amount of wear for all of the coatings without exposing the steel substrate underneath. In this way, the wear rates and chemical properties of the coatings could be compared without any influence from the substrate. Experiments were always conducted three times. Humidity was kept constant throughout the experiment by flowing humidified air at a specific rate through the test chamber. Full experimental settings are given in Table 2.

**Table 2:** Experiment Settings for the Tribological Experiments

ball diameter	10 nm
normal force	10 N
experiment duration	5 min
frequency	10 Hz
stroke length	1 mm
humidity	50 ± 5% r H
temperature	ambient
lubricant	none

### Surface Characterization

The topographies of the wear tracks were obtained by using atomic force microscopy (AFM) with a Veeco Dimension V system (from Veeco Instruments, Plainville, USA). Data was acquired in tapping mode over an area of 20 × 20 μm^2^, with a resolution of 256 × 256 and 512 × 512 pixel for the undoped and B-doped coatings, respectively.

The surfaces were characterized before the tribological tests by white light interferometry to evaluate the initial surface roughness. This analysis is described in the Supporting Information.

The surface chemistry of the ta-C:B coating was investigated by X-ray photoelectron spectroscopy (XPS). The results, which are shown in the Supporting Information, indicate the presence of a thin oxidation layer on the surface of the coating both inside and outside the wear track. Furthermore, the distribution of B in the bulk material is uniform and matches the value obtained by EDS. No significant differences in the surface chemistry were observed between the wear track and the pristine surface.

Raman spectra of the surface of the coatings, also shown in the Supporting Information, indicate that the coatings with similar hardness are also structurally similar, with the softer materials presenting a mild structuring of sp^2^ carbon.

### Wear Depth and Area

The wear depth and area were measured by using a contact profilometer (Accretech, Tokyo, Japan). The tip’s measuring length was 1000 μm with a speed of 0.060 mm/s. All experiments were performed in the center of the wear track, orthogonal to the sliding direction. From the resulting profile, the maximum wear track depth and cross-sectional area were calculated.

### Estimate of the Contact Shear Stress

The quantum-chemical molecular dynamics simulations presented in ref (12) provide a connection between the average shear stress at asperity-asperity contacts and the chemical state of diamond–diamond sliding interfaces (e.g., H/OH passivation, various cold-welding regime characterized by interfacial covalent bonds). To estimate which friction regime (or the chemical state of the interface) is responsible for the experimentally measured friction coefficients, a qualitative estimate of the average contact shear stress is necessary.

The average local contact shear stress can be defined as τ = *F*_L_/*A*_R_,^45^ where *F*_L_ is the lateral (friction) force and *A*_R_ is the real contact area. Moreover, *F*_L_ = μ*F*_N_, where μ is the measured friction coefficient and *F*_N_ is the applied normal force. Persson’s contact theory^46,47^ can be used for a qualitative estimate of the real contact area *A*_R_. It was recently shown that this theory can describe the ordering of the average local pressure, in agreement with contact mechanics simulations^48,49^ and can provide a qualitative estimate of its values for contacts between a variety of DLC coatings.^2^ Moreover, the parameter E*h′_RMS_ was useful to rationalize the experimental results in terms of the elastic properties and the surface roughness of the coatings. Here, E* is the contact modulus, while *h*′_RMS_ is the composite root-mean-square (RMS) slope of the contacting surfaces. The contact modulus is obtained from 1/E* = (1 – ν_1_^2^)/*E*_1_ + (1 – ν_2_^2^)/*E*_2_, where ν_1_, ν_2_, *E*_1_, and *E*_2_ are the Poisson’s ratios and Young’s moduli of the two contacting materials, respectively. The Young’s moduli of the amorphous carbon films are reported in Table 1, and a Poisson’s ratio of 0.19 is assumed for all the coatings.^50^ Concerning the alumina ball, a Young’s modulus of 400 GPa and a Poisson’s ratio of 0.27 were used, according to the data provided by the supplier.

The composite RMS slope *h*′_RMS_ is obtained from the AFM surface topographies of the two contacting surfaces as . The RMS slopes of the individual surfaces were calculated as^51^
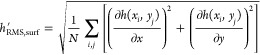
1where ∂/*∂x* and ∂/*∂y* are discrete derivatives in the *x* and *y* directions calculated using finite differences, *h*(*x*_*i*_, *y*_*j*_) is the AFM discrete height map, and *N* is the number of data points in the height map. All the topographies were obtained in the wear scar since only the roughness of the worn surfaces can be related to the friction coefficient at a steady state. The initial surface roughness of the lapped coatings, which is relevant for the running-in behavior, was estimated by white light interferometry, and the results are presented in the Supporting Information. For ta-C, ta-C:B, and a-C:B, three different pairs of topographies were considered for the disc and the ball for a total of 18 maps. For a-C, only two pairs of maps were obtained. Therefore, one additional pair of topographies was generated by matching the ball of the first pair with the disc of the second pair. The AFM topographies are included in the Supporting Information.

Using these parameters, within the framework of Persson’s contact theory, the real area of contact can be expressed as
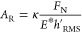
2where . Hence, τ = *F*_L_/*A*_R_ = μ*F*_N_/*A*_R_ can be calculated as
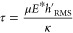
3Within the same theory, the median effective contact pressure can be calculated as follows:^2^
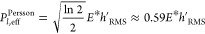
4

### Tight Binding Simulations

A series of tight binding (TB) simulations were used to calculate the critical contact normal pressure above which interfacial bonds form between a passivated and an unpassivated a-C surface as a function of the boron content. The simulations were performed with the extended tight-binding Hamiltonian developed by Grimme and co-workers, called GFN1-*x*TB,^52^ implemented in the DFTB+ software package.^53^ Amorphous carbon bulk samples with a cell size of 10 × 10 × 12 Å^3^ and a density of 2.9 g/cm^3^ were first prepared by rapidly quenching a melt from 5000 to 0 K. A slab geometry was created by cutting the bulk sample perpendicular to the *z*-axis at a given *z*-coordinate and introducing a vacuum region spanning 38 Å along the *z*-axis. A tribo-pair was then prepared by placing two slabs into a 10 × 10 × 50 Å^3^ simulation cell.

The dangling bonds of the outermost ta-C layers were terminated with hydrogen atoms. Six hydrogen atoms and six hydroxyl groups were added in order to terminate dangling bonds on the inner surface of the lower slab, whereas no terminating species were added to the upper slab. The simulations mimic a situation that is critical for friction, in which during an asperity-asperity contact, one of the surfaces is passivated by the H/OH products of water dissociative chemisorption, while the other surface is depassivated owing to a previous asperity collision.

The effect of B-doping on the critical pressure for interfacial bond formation was investigated by replacing randomly chosen surface carbon atoms of the upper slab with B atoms. After geometry optimization, quasi-static pressurization simulations were performed. In the quasi-static simulations, the outermost layers of both slabs were kept rigid, and the rigid layer of the upper slab was moved toward the lower slab along the *z*-axis in steps of 0.2 Å. For each step, the positions of all atoms except for those of the rigid layers were relaxed until all force components were lower than 1 × 10^–2^ eV/Å. The normal pressure at each step was calculated by dividing the sum of the force components along the *z*-axis for all atoms in the rigid layer of the upper slab by the lateral area. The critical pressure for the formation of interfacial bonds was defined as the normal pressure that is required for the formation of the first covalent bond across the interface; that is, the normal pressure at the step just before the bond-formation reaction occurs. The quasi-static pressurization simulations were performed with a number of B atoms varying from 0 to 4, hereafter indicated by 0B–4B. Eight 0B (pure ta-C) systems with different ta-C structures were first prepared, and then, random substitutions were carried out to obtain the 1B–4B systems. In total, 40 quasi-static pressurization simulations were performed.

### Density Functional Theory Simulations

DFT simulations were carried out using the Quantum ESPRESSO computational suite,^54^ following the plane-waves and pseudopotentials approach. The PBE exchange-correlation functional^55^ implemented in the Ultrasoft GBRV pseudopotentials^56^ was used. The cutoff values for the kinetic energy of the plane waves and the charge density were 30 and 240 Ry, respectively. Integration of the charge density was carried out on a 2 × 2 × 1 Γ-centered K-point grid. Spin polarization was considered in all of the simulations, and a Gaussian smearing of 0.002 Ry was adopted for the electronic states around the Fermi level. Grimme’s DFT-D2 correction^57^ was used to describe long-range interactions.

The diamond (100) and (111) surfaces (labeled as C(100) and C(111) hereafter) were modeled by slabs composed of 12 and 8 carbon layers along the *z*-axis, respectively. The bottom surfaces of each slab were terminated with H atoms. While the top surface of C(100) was reconstructed by (2 × 1) C–C dimers, C(111) was left unreconstructed. The lateral dimensions of the simulation cells, namely, 9.98 × 7.61 Å^2^ and 8.73 × 7.57 Å^2^ for C(100) and C(111), respectively, were obtained by replicating the unit cell 4 and 3 times along the *x* and *y* directions, respectively, and by performing a variable-cell geometry optimization using the BFGS algorithm. The convergence thresholds for the force and stress components were 10^–3^ Ry/bohr (∼2.57 × 10^–2^ eV/Å) and 5 × 10^4^ Pa, respectively. The height of the simulation cells was chosen to ensure at least 14 Å of vacuum in the vertical direction.

The adsorption energy of water and methanol on the diamond surfaces was calculated as
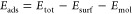
5where *E*_tot_, *E*_surf_, and *E*_mol_ are, respectively, the total energies of the adsorbed system, of the surface alone, and of the isolated molecule in the same simulation cell of the adsorbed system. The latter was compared to the energy of a single molecule in a cubic cell with an edge of 30 Å using the computational parameters described above, except for the sampling of the Brillouin zone, which was carried out at the Γ point. In the simulation cell of the adsorbed system, the resulting interaction energies of the molecules with their periodic replicas never exceeded 8 meV.

Quasi-static pressurization simulations were performed by placing the top slab at an initial interfacial distance of approximately 9 Å. The top slab was then moved toward the bottom surface in steps of 0.2 Å. The geometry of the system was optimized at each step while keeping the position of the top and bottommost carbon atoms fixed.

The geometry of α-Al_2_O_3_(0001) slabs was optimized in orthorhombic cells with a size of 8.23 × 4.75 × 25.00 Å^3^. A single layer of aluminum atoms was exposed on the unterminated surfaces of the slabs, which were composed of 40 atoms in total (corresponding to four complete Al–O_3_–Al layers). In the calculations involving amorphous carbon, the size of the Al_2_O_3_ slab was doubled in the *y* direction. The width of the Gaussian smearing was increased to 0.02 Ry to facilitate convergence of the self-consistent calculations. The C6 coefficient of Grimme’s DFT-D2 parametrization for Al was reduced to the corresponding value for Ne (21.855 Ry × bohr^6^ = 0.63 J × nm^6^/mol) since the interaction energy of the metallic cations is generally overestimated using the original parameters.^58^

Two amorphous carbon samples were generated following a melt-quench protocol^59^ using the LAMMPS code^60^ and the REBO2 potential.^61^ A Langevin thermostat with a time constant of 0.1 ps was used, and the time step was set to 0.1 fs. First, a random distribution of 392 C atoms was equilibrated at 5000 K for 20 ps. The lateral dimensions of the simulation cell were the same as those for the large Al_2_O_3_ slab, and the cell height was 40 Å, leading to a density of approximately 2.5 g/cm^3^. The system was then quenched at a constant rate to 300 K for 20 ps. After a geometry optimization using the FIRE algorithm,^62^ the system was cut at two different heights to generate a slab of approximately 14 Å in thickness. Undercoordinated surface atoms resulting from the cut were saturated by H atoms except for the bottom surface of the top slab in the DFT quasi-static simulations. The geometry of the amorphous carbon slabs was optimized using Quantum ESPRESSO with the corresponding computational parameters described earlier, except for the width of the Gaussian smearing, which was chosen to be 0.02 Ry to match the value selected for Al_2_O_3_. An OH group was placed on the sp^2^-C atom of the amorphous carbon surface, which was substituted by B for the doped case. The geometries of all the simulated systems were visualized and analyzed with OVITO.^63^

## Results

### Experimental Results and Estimate of the Contact Shear Stress

**Figure 1:**
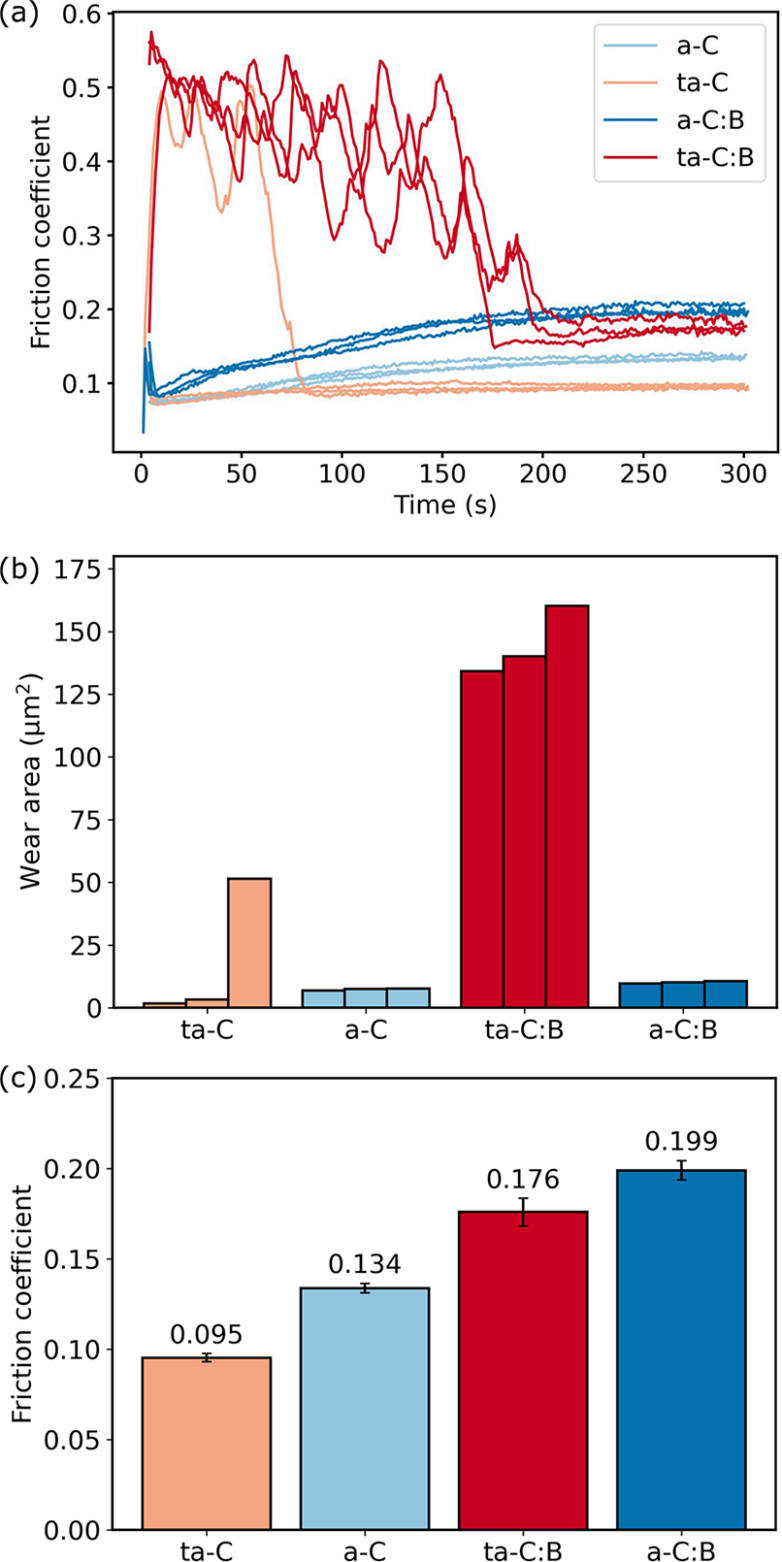
(a) Results of the ball-on-disc tribological tests on the undoped and B-doped a-C and ta-C samples. (b) Wear areas measured for all of the tribological tests. The maximum wear depth is shown in the Supporting Information, along with the numerical values of the wear areas. (c) Average friction coefficients during the last 75 s of the three test runs for each type of coating. The error bars represent the standard deviation of the friction coefficients calculated in the same range.

Figure 1 shows the results of three independent tribological tests for each coating. All of the tests performed on the ta-C:B coating are characterized by a severe running-in phase, which lasts approximately 200 s. A shorter running-in time is also observed in one of the three tests for the undoped ta-C. After this initial phase, the friction coefficients of the undoped and B-doped ta-C films reach approximately 0.10 and 0.18, respectively. The friction coefficient obtained with the a-C coatings stays relatively low during the whole tests, despite a slight increase, reaching values of 0.13 and 0.20 for the undoped and B-doped case, respectively.

The high friction coefficient observed during the running-in period of ta-C:B and in one of the tests on the undoped ta-C is related to high wear, as shown in Figure 1b, and might be caused by their high hardness, which translates into higher contact pressures than in the a-C and a-C:B cases for the same roughness. High local pressures can cause surface depassivation and interface cold welding, leading to high friction and wear.^13^ Subsequent surface smoothing can cause a drop in the local contact pressure so that a steady state with relatively low friction is reached. However, the fact that not all ta-C coatings undergo a severe running-in like in the ta-C:B case and that the duration of the running-in for the undoped ta-C is considerably shorter than for the ta-C:B coatings might indicate that the presence of B affects the stability of their surface passivation. This facilitates the formation of covalent bonds across the sliding interface and, hence, cold welding. The same argument might be used to explain why the B-doped coatings show higher steady-state friction coefficients in comparison to their undoped counterparts. This can be caused by the occasional formation of covalent bonds across the sliding interface of the B-doped systems. Therefore, we hypothesize that the tribological properties of the boron-doped coatings can be explained by an increased tendency to form covalent bonds across the sliding interface.

While the validation of this hypothesis is the main goal of the quantum-mechanical simulations presented in the next sections, it is useful to estimate whether the experimentally measured friction coefficients are compatible with a change in the friction regime caused by B-doping. The systematic simulation study presented in ref (12) provides the possibility to relate local contact shear stress at asperity contacts to the chemical structure of the carbon–carbon sliding interface, that is, the friction regime. Using the experimental values of the friction coefficients presented in Figure 1, the AFM surface topographies, and Persson’s contact theory, as explained in the Materials and Methods section, the average contact shear stress τ was estimated. Its values for the four tribosystems considered in this study are listed in Table 3 together with the parameters used to estimate them: the composite RMS surface slope *h*′_RMS_, the contact modulus E*, and the product E*h′_RMS_, which within Persson’s contact theory is proportional to the median effective contact pressure (details in Materials and Methods).

**Table 3:** Calculated Contact Parameters of the Coatings Based on Experimental Conditions

coating	RMS slopes *h*′_RMS_	contact modulus E* (GPa)	E*h′_RMS_ (GPa)	shear stress τ (GPa)
**ta-C**	0.040 ± 0.001	259.40	10.27	0.61
**a-C**	0.054 ± 0.003	197.78	10.76	0.90
**ta-C:B**	0.092 ± 0.010	250.73	23.11	2.55
**a-C:B**	0.131 ± 0.004	157.04	20.57	2.57

The results of this estimate show that the ordering of the contact shear stress values follows that of the friction coefficients in Figure 1. Moreover, there is a clear separation between undoped and B-doped coatings in terms of E*h′_RMS_ and τ values, which appear to depend on doping rather than on the type of coating (a-C or ta-C). To be able to fairly compare these contact shear stress values with those calculated by means of tight binding simulations in ref (12), an estimate of the average local contact pressure for each tribosystem is necessary. Eq 4 yields 6.0 and 6.3 GPa for ta-C and a-C as well as 13.6 and 12.1 GPa for ta-C:B and a-C:B, respectively. Ref (12) shows that, for self-paired diamond interfaces, values of τ below 1 GPa at a normal contact pressure of 5 GPa are characterized by sliding interfaces that are fully passivated by H/O atoms or OH groups. Given the similar average local contact pressures estimated for the undoped a-C and ta-C coatings, these results suggest that these sliding interfaces are fully passivated. The estimated local contact pressure values for ta-C:B and a-C:B are a factor of 2.2 and 1.9 larger than the ones of ta-C and a-C, respectively. Since the local shear stress varies linearly with normal pressure for H/OH-passivated carbon interfaces,^13^ one would expect shear stress values lower than 1.8 GPa for the B-doped coatings if their interfaces were fully passivated. Instead, the τ values of the ta-C:B and a-C:B coatings are approximately 2.6 GPa. These higher values hint toward the occasional formation of interfacial covalent bonds. Indeed, assuming a weak dependency of the local shear stress on normal pressure, these values can be ascribed to a mild cold-welding regime characterized by the formation of interfacial ether bridges, which show shear stress values ranging from about 1 to 3 GPa in ref (12).

Despite its qualitative significance, this estimate confirms the plausibility of the hypothesis formulated earlier. That is, the presence of B on the surfaces of the coatings somehow facilitates the occasional formation of interfacial bonds that result in an increase in friction. The goal of the quantum-mechanical simulations presented below is to investigate which tribochemical mechanisms may trigger this surface depassivation process.

### Formation of Interfacial Bonds: TB Simulations

In the first simulation step, TB simulations are used to investigate whether the presence of B atoms on the coating’s surfaces can facilitate the formation of interfacial bonds, which would explain the change in the friction regime discussed above. Since modeling amorphous materials require a large number of simulations to obtain statistically meaningful results, the TB method is used instead of DFT owing to its efficiency and accurate description of carbon systems.^2,12,13,64,65^ The results will be later validated, rationalized, and generalized to other interfaces by means of DFT simulations.

The model system chosen for the TB simulations is a representative volume of an asperity-asperity contact between two amorphous carbon surfaces. Here, it is assumed that a carbon transfer film forms on the alumina counter surface during running-in, as often observed when DLC coatings are paired to other materials.^9,66−68^ The case in which the transfer film does not occur and the sliding interface is located directly between the DLC surface and the alumina ball will be investigated by DFT simulations and discussed in the following sections. In a humid environment, surface dangling bonds are usually passivated by H and O atoms or OH groups. Previous TB molecular dynamics simulations^12,13,65^ show that when two passivated carbon surfaces slide against each other, no chemical reactions occur for the whole simulation time (∼1 ns) even at local normal pressures as high as 10 GPa. However, during asperity-asperity contacts, plastic or fracture processes can lead to surface depassivation,^69^ which makes at least one of the two contacting surfaces temporarily reactive. These passivation and repassivation processes occur repeatedly. To mimic this critical situation, in the model system considered here, one of the two surfaces is fully passivated with H atoms and OH groups, while the other one is left unpassivated.

**Figure 2:**
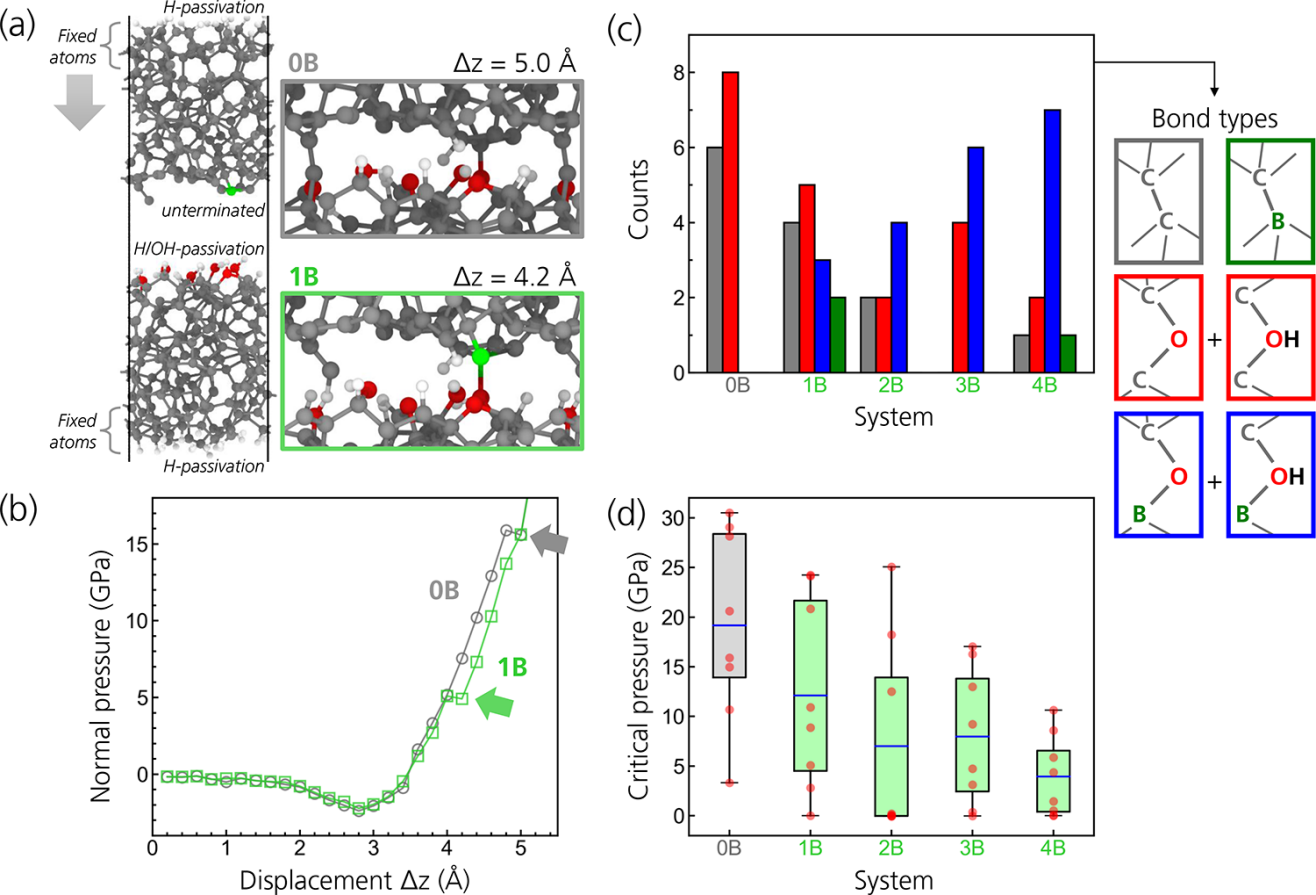
TB quasi-static pressurization simulations of undoped and B-doped a-C interfaces. (a) Lateral views of a representative B-doped interface summarizing the computational setup (left). Snapshots of the undoped (0B) and B-doped (1B) interfaces at different displacement values Δ*z* show the formation of the first interfacial bonds (right). (b) Normal pressure as a function of Δ*z*. The pressure is calculated as the sum of the vertical components of the atomic forces on the topmost constrained atomic layer divided by the area of the simulation cell. The formation of the first interfacial bonds is characterized by the slight drops in pressure at ∼5 and ∼16 GPa highlighted by the gray and green arrows. (c) Total occurrence of different types of interfacial bonds during 40 quasi-static TB simulations (8 ta-C systems for each B concentration). (d) Distribution of the critical pressures in the quasi-static simulations. The blue horizontal lines and boxes indicate the means and standard deviations of critical contact pressures, respectively. The ends of the whiskers represent the minimum and maximum critical contact pressures for each system.

As a first example, one quasi-static simulation is presented to illustrate the interface chemical processes in undoped and B-doped systems. To calculate the critical contact pressure at which interfacial covalent bonds form across the interface between carbon surfaces, the unpassivated top slab was gradually displaced toward the passivated bottom slab, as shown in Figure 2a (see Materials and Methods for details). The contact pressure in this simulation setup is controlled indirectly through the displacement of the top slab. The undoped system is compared with a B-doped system obtained by replacing a single surface C atom of the undoped top slab with B. No significant changes in the bulk structure of the B-doped slab were observed after structural relaxation, and the substitution only affected local atomic structures.

The evolution of the normal pressure as the upper slab moves quasi-statically toward the lower slab (increasing displacement) is plotted in Figure 2b (gray and green lines for the undoped and B-doped systems, respectively). Initially, the pressure is zero as the surfaces do not interact with each other. The pressure evolution is initially identical for both systems. As the two slabs approach each other, the pressure reaches a minimum negative value and then starts increasing as the two slabs repel each other. At a displacement of Δ*z* = 4.2 Å and a critical pressure of 5.1 GPa, the pressure in the doped system drops slightly and the curve shows a discontinuity as the first interfacial covalent bond forms. This is a B–(OH)–C bridge (bottom right snapshot in Figure 2a). In contrast, the pressure for the undoped system continues to increase until the two interfacial bonds form at Δ*z* = 5.0 Å and the corresponding critical normal pressure is 15.9 GPa. It is worth noting that this value is much higher than the average contact pressure estimated in the experiments shown here for undoped systems (about 6 GPa). The high contact pressure leads to the cleavage of the O–H bond of a terminating OH groups and the subsequent formation of a C–O–C bond across the interface, along with an interfacial C–C–C chain.

This example shows how the presence of a single B atom in the slab (the concentration in the slab is 0.4 at %) can lead to the activation of an OH passivation group and significantly lower the critical contact pressures at which covalent bonds form across the interface. To give statistical significance to this result, the same kind of simulation was performed with 8 different ta-C interfaces. For each interface, 5 different B concentrations (0B–4B) were considered by replacing C atoms with B atoms in the near surface region (details in Materials and Methods). The results are summarized in Figure 2c,d.

Figure 2c shows the sum of the occurrences of different kinds of interfacial bonds for the five different B contents. When boron is absent (0B), the interfaces were generally cold weld by forming C–C and C–O–C bonds. Only a minority of the interfacial bonds consist of C–OH–C bonds, since oxygen is overcoordinated and tends to donate the H atom to a neighboring C site. For the B-doped systems, C–O–C, C–OH–C, B–O–C, and B–OH–C are overall dominant over C–C and C–B interfacial bonds. Moreover, the number of B–O–C and B–OH–C bridges increases with increasing B content. In this case, the bridging OH group without cleavage of the O–H bond is stable since oxygen forms two covalent bonds with C and H and a dative bond with B.

Figure 2d shows the distribution of the critical pressures to achieve interface bonding for the five B contents. The average critical pressure decreases with increasing B concentrations at the interface. While the average critical pressure for the 0B systems is almost 20 GPa, that is, significantly higher than the estimated average contact pressure in the experiments with undoped coatings, the 2B, 3B, and 4B systems have critical pressures that are lower than the ∼13 GPa pressure estimated for the experiments with B-doped coatings. Overall, these results confirm that the presence of B atoms at the sliding interface facilitates the formation of interfacial bonding, which leads to an increase in the interface shear stresses. Moreover, the TB simulations suggest that the depassivation of OH surface groups can be attributed to the characteristic chemistry of boron, which can act as an electron-pair acceptor and form a dative bond with the oxygen atom of the surface OH groups. To further investigate this mechanism and its generality, DFT simulations were preformed and are the subject of the following section.

### Effect of the B–O Dative Bond on the Interface Passivation: DFT Simulations

To begin an in-depth study of the interface depassivation mechanism based on B–O dative bonds, DFT simulations were performed on well-defined model systems that enable the rationalization of the mechanisms at play. In a first step that illustrates the nature of the B–O dative bond, the adsorption of water on crystalline surfaces, namely, diamond (100) and (111), is studied. The main goal of these simulations is to examine in detail the difference between water adsorption on undoped and B-doped surfaces and, in particular, to illustrate the difference between the C–O and the B–O bonds.

**Figure 3:**
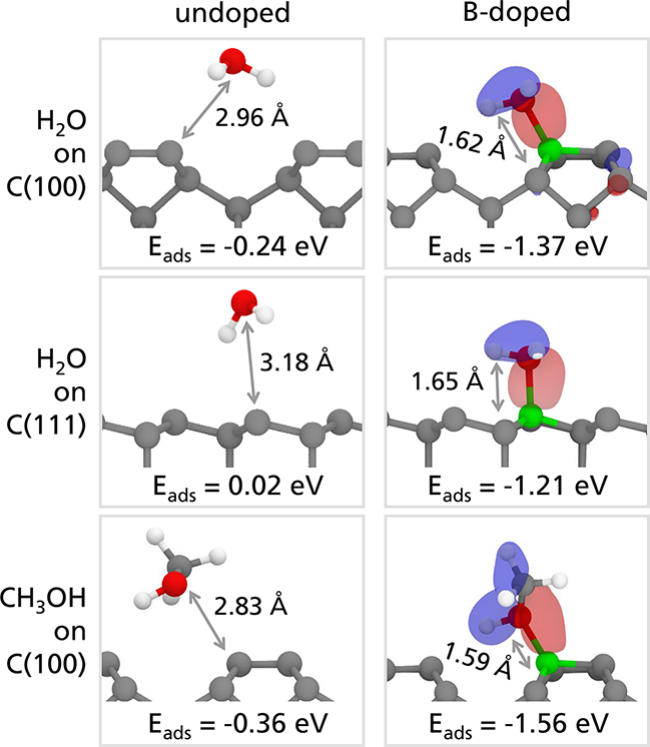
Optimized geometries of water on the undoped and B-doped C(100) and C(111) surfaces and of methanol on the C(100) surface. White, green, gray, and red spheres represent hydrogen, boron, carbon, and oxygen atoms, respectively. In the right panels, the molecular orbital associated with the B–O dative bond is shown. The isosurfaces were obtained by plotting the sum of the spin-up and spin-down contributions to the wave function using an isovalue of 0.005 1/bohr^3^. The red and blue colors of the isosurfaces represent the positive and negative sign of the wave function, respectively.

Water can either physisorb or undergo dissociative chemisorption on undoped C(100) and C(111) surfaces (Figure 3). However, the presence of boron enables an additional chemisorbed but undissociated state, as shown in Figure 3. This adsorbed state is characterized by adsorption energies of −1.37 and −1.13 eV for the C(100) and C(111) surfaces, respectively. These energies, as well as the B–O bond lengths, which are significantly shorter than the equivalent C–O distances in the undoped case, are in agreement with previous DFT calculations.^37^ This configuration is made possible by the dative bond that forms between B and O. That is, oxygen shares one of its electronic lone pairs with B, while it maintains two stable covalent bonds with the H atoms. For each of the chemisorbed configurations on the B-doped surfaces, the molecular orbital associated with the B–O dative bond is shown in Figure 3. These orbitals are found at approximately 9.7 eV below the Fermi level, and their strongest contributions are the *p* atomic orbitals of the O and B atoms.

This adsorption mechanism is not exclusive to water. An example for another simple OH-containing molecule, that is, methanol, on the C(100) surface is shown in the bottom row of Figure 3. The length and the energy of the dative bond formed between the B atom of the surface and the O atom in methanol (1.59 Å and −1.56 eV) are similar to the ones of water (1.62 Å and −1.37 eV), indicating that the B–O dative bond can form regardless of the atoms surrounding the O atoms. For instance, OH groups on an amorphous carbon countersurface can react in a similar way. These groups, that generally passivate the dangling bonds of diamond and amorphous carbon surfaces, can be activated by boron and form B–OH–C bridges across the interface, as demonstrated by the mild interface cold-welding regime observed in the TB simulations. Moreover, the short distances between the molecules and the B-doped surfaces suggest that a tribological countersurface can get closer to the B-doped substrate than in the undoped case, with an effect on friction.

To proceed this investigation further, the effect of B-doping on the stability of OH passivation at diamond interfaces is considered next. Two additional sets of simulations were performed, inspired by the TB pressurizations described in previous sections. In the first simulation set, the geometry of self-mated C(100) interfaces and of unreconstructed and Pandey-reconstructed C(111) interfaces was optimized without any constraint. In the case of C(100), a H/OH pair, representative of the dissociated state of one water molecule, was placed on a surface C–C dimer. For C(111), an OH group was placed on the bottom surface. In the case of the unreconstructed C(111) interface, H atoms were used to passivate all of the remaining C atoms of the bottom surface to prevent the spontaneous cold welding with the top surface. In all cases, the top slab was initially placed symmetrically on top of the bottom slab.

**Figure 4:**
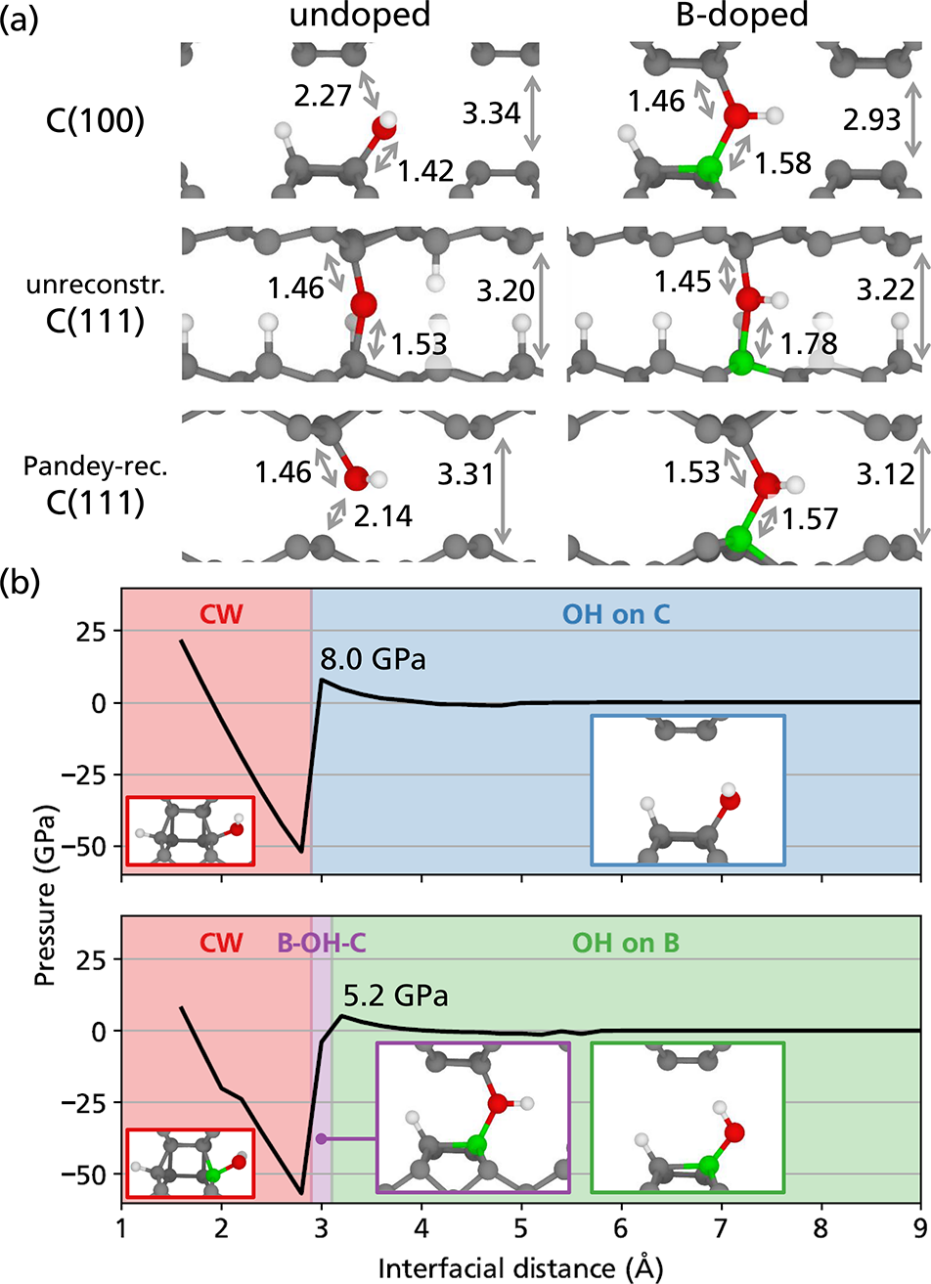
OH group at the interface of undoped and B-doped C(100) and C(111) surfaces. (a) Optimized geometries of the interfaces. All of the bond lengths are expressed in Å. (b) Stability diagrams of the OH group at the undoped and B-doped C(100) interfaces. The labels CW, B–OH–C, and OH on C and OH on B indicate cold welding, the presence of a B–OH–C interfacial bridge, and a stable OH group attached to a C or B surface atom, respectively. The value of the critical pressure before the formation of interfacial bonds is also included in the diagrams.

Figure 4a shows the optimized geometries of these interfaces. As expected, the interfacial distance of B-doped C(100) turned out to be ∼0.4 Å shorter than in the undoped case, owing to the presence of a B–OH–C bridge. The length of the C–O bond in the B–OH–C bridge is compatible with that of a single covalent bond. In the case of undoped C(111), the bond lengths and the interfacial distance of this latter system are similar to those of the B-doped case. Nevertheless, the OH group decomposes further, releasing the H atom on the top surface, while in the B-doped interface, the OH group remains intact and forms a stable B–OH–C bridge. The results for the Pandey-reconstructed C(111) interface, which is significantly less reactive than unreconstructed C(111), are similar to those for the case of C(100).

In a second set of simulations, a quasi-static pressurization of the C(100) interface was performed starting from a similar geometry as in the previous simulation, with the top surface at approximately 9 Å from the bottom surface. The procedure was the same as in the TB simulations: the top slab was gradually displaced toward the bottom slab, and the pressure experienced by the constrained atoms was calculated at each step (details in Materials and Methods). The critical normal pressure reached before the formation of interfacial bonds turned out to be 5.2 and 8.0 GPa in the B-doped and the undoped diamond surfaces, respectively, as shown in Figure 4b. Different regions describing the state of the interface are highlighted in the plots in the figure. At large interfacial distances, the OH terminations stay intact up to the critical pressure, while at small interfacial distances, the system cold-welds. These regions can be found for both the undoped and B-doped interfaces. For the B-doped system, an additional region can be observed, corresponding to a stable B–OH–C bridge across the interface at an interfacial distance of 3.0 Å. A similar C–OH–C bridge cannot be observed in the undoped system since the interfacial C–C bonds are formed before any rearrangement of the OH group, which is repelled by the electronic density of the top surface.

**Figure 5:**
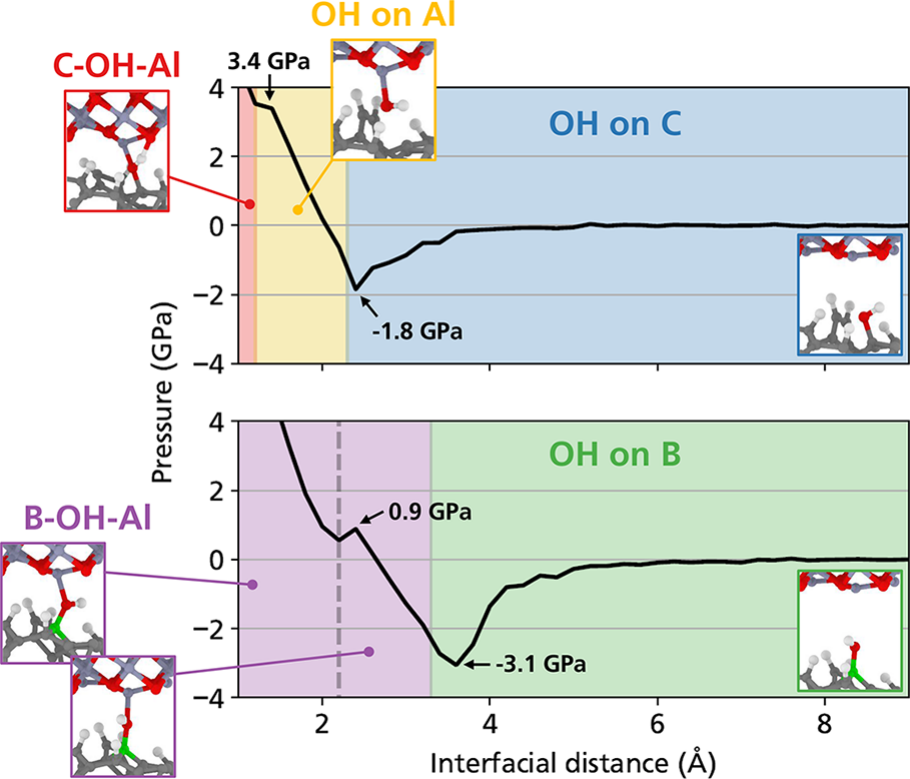
Stability diagrams of OH at the undoped and B-doped a-C/Al_2_O_3_ interfaces.

Even though the calculations described so far offer a clear indication of the role of the B–O dative bond in promoting the formation of interfacial bonds, one final issue needs to be addressed, namely, the possibility of observing this mechanism for material pairings other than carbon–carbon. While the transfer of a carbon film on the surface of the Al_2_O_3_ ball used in our experiments is likely, as observed when pairing (hydrogenated) DLC with silicon nitride^9,66^ or Al_2_O_3_,^70^ it is useful to consider the case in which this transfer film is not present. For this reason, additional quasi-static pressurization simulations with DFT were performed for an interface between amorphous carbon (top slab) and Al_2_O_3_ (bottom slab). One of the topmost sp^2^-hybridized C atoms of the bottom surface, which was replaced by B in the doped case, was chosen as the adsorption site of an OH group, while all of the other undercoordinated C atoms were saturated by H atoms. The stability diagrams of these simulations are shown in Figure 5.

During the pressurization simulations, the OH group is attracted to the Al atoms of the top slab, resulting in a negative pressure in the plots in Figure 5. After reaching −1.8 GPa, the OH group desorbed from a-C and attached to the Al atom above. This exchange was not observed in the B-doped case, where the OH group remained attached to B, and the pressure reached −3.1 GPa. After this point, a B–OH–Al interfacial bridge formed. The OH group in the undoped case became unstable upon further pressurization with the H atom shared between the two nearest O atoms, while the B–OH–Al bridge remained stable until the end of the simulation. These results indicate that the formation of OH-mediated interfacial bonds due to the presence of B–O dative bonds can be valid also for different material pairings in which the countersurface does not contain carbon.

## Discussion

In summary, the quantum-mechanical simulations performed in this study show that even in the case of partial H/OH surface passivation, no covalent interfacial bond forms at undoped carbon–carbon interfaces for local contact pressure values typical of the tribological experiment. The presence of B atoms on the carbon surfaces decreases the critical local normal pressure for the formation of interfacial bonds to values that are lower than those estimated for the ball-on-disc experiments presented here. The surface depassivation mechanism relies on the ability of B atoms to bind to otherwise unreactive OH surface terminations via a B–O dative bond. The resulting C–OH–B interface bridges, which are responsible for shorter interfacial distances compared to the undoped case at the same contact pressure, can easily transform into C–O–B bridges upon tribologically induced H transfer. While this situation corresponds to the experimental conditions in the case of a carbon transfer film formation on the alumina ball, the simulations also show how Al–OH–B interface bonds are stable at the interface between amorphous carbon and alumina, suggesting the transferability of this mechanism to hybrid interfaces.

This effect of boron doping on the tribochemistry and friction regime of amorphous carbon coatings is fully in line with the scenario outlined on the basis of the experimental results and the contact-theory analyses presented above. The tribological tests show that B-doping leads to an increase of friction: from 0.095 to 0.176 for ta-C and from 0.134 to 0.199 for a-C. To relate these experimental results to the chemical state of the interfaces (friction regimes) provided by the simulations, values of local contact shear stress and local normal contact pressure were estimated applying Persson’s contact theory to surface topography measurements on the wear scars. The estimate suggests that the friction coefficients obtained with undoped coating are compatible with sliding at a passivated interface. Instead, the friction coefficients obtained with B-doped coatings are compatible with shear stress values typically obtained in the presence of a mild cold-welding regime in which sliding is characterized by the formation and breaking of a sparse network of ether-like interfacial bridges.

Despite the consistent scenario in which experiments and simulations merge, it should be noted that, in general, the effect of doping can be diverse and has multiple consequences on friction and wear. Several aspects of the tribology of B-doped coatings still require further investigation. The mechanical properties and the surface topographies of the films, which cannot be taken into consideration in our atomistic simulations, play a crucial role in determining the friction performance in the initial phase of the tribological tests. The enhanced electronic conductivity and the improved oxidation stability due to B-doping might also alter the surface reactivity and the surface reconstructions with an impact on friction. Indeed, reports on the effect of B-doping on the tribological properties of carbon coatings are diverse and sometimes contradictory. For instance, some studies indicate that the presence of B in the carbon material is beneficial to reduce friction and wear.^19,23,24^

Nonetheless, the tribochemical effect of B-doping described in this work may be relevant for various applications of carbon coatings. The experiments described in this work are relevant to the use of amorphous carbon coatings in tribological applications under a high load in a humid environment. In this context, doping with boron can worsen the friction and wear properties. However, the effect may be counterbalanced by other benefits described in the literature such as improved surface quality^41,43^ and oxidation resistance.^27,28^ Under more severe conditions, such as those typical of cutting tools, the increase in surface reactivity due to boron doping is certainly less relevant than other effects induced by the presence of boron. Indeed, B-doping significantly increases the wear resistance of diamond cutting tools.^18^ This effect is not only due to changes in mechanical and chemical properties of the material but also to the increase in electrical conductivity, which prevents damage by triboelectric plasma discharge.^18^

A field of tribology in which boron’s affinity for O and OH is potentially very interesting is that of superlubricity applications. In this field, DLC coatings are often used in combination with OH-containing, organic friction modifiers (e.g., oleic acid, glycerol) to achieve friction coefficients lower than 0.01.^64^ Since superlubricity in these applications is triggered by the chemisorption of these friction modifiers on the DLC surface, B-doping could be exploited to tune the chemisorption process via dative bonds. Similarly, the peculiar way in which O-containing molecules bind to B on carbon surfaces could influence the surface chemistry and environmental response of carbon materials used in applications fields other than tribology. For example, B-doped crystalline and amorphous carbon materials are also widely employed for electrochemical and electroanalytical applications because of their enhanced conductivity, combined with their chemical inertness and biocompatibility, making these materials particularly suited to build electrodes.^32^

## Conclusions

Using a scale-bridging approach, which combines tribological tests, surface roughness measurements, contact theory, and atomistic simulations, this work uncovered a tribochemical contribution of boron doping to the tribological properties of carbon coatings. Estimates of the average contact stress based on the theory of contact, and on experimental measurements of the coating’s surface topographies and their elastic moduli, suggest that the friction increase has a chemical rather than a mechanical origin. The estimated average shear stress values are typical of a surface passivation regime in the case of undoped coatings, while they are compatible with the formation of interfacial ether-like bonds for the B-doped coatings. Quantum-mechanical simulations confirm that at contact pressures compatible with the experimental ones, the presence of B on the coatings’ surfaces can cause surface depassivation via formation of B–O dative bonds that, in turn, lead to the formation of ether-like interfacial bridges.

In general, the results discussed in this paper emphasize how even fine, localized chemical effects caused by the presence of small amounts of impurities on surfaces or at materials interfaces can have important effects on macroscopic quantities like friction and wear. These chemical details cannot be overlooked when designing surfaces and interfaces with an increasingly optimized functionality.

## Supporting Information Available

Roughness estimation, wear measurements, AFM topographies, and XPS and Raman analyses (PDF)

## Abbreviations

a-C: amorphous carbon
ta-C: tetrahedral amorphous carbon
DLC: diamond-like carbon
PVD: physical vapor deposition
QCSM: quasi-continuous stiffness method
AFM: atomic force microscopy
RMS: root-mean-square
TB: tight-binding
DFT: density functional theory.
