# Supplementary material for: Surface Depassivation via B–O Dative Bonds Affects the Friction Performance of B-Doped Carbon Coatings
Source: ACS Appl Mater Interfaces. 2024 Mar 28;16(14):18112–23. doi: 10.1021/acsami.3c18803 (PMC11011640; doi:10.1021/acsami.3c18803)
Supplement: Supplementary file 1 — am3c18803_si_001.pdf [file am3c18803_si_001.pdf]

Supporting Information

Surface depassivation via B–O dative bonds affects the friction performance of B-doped carbon coatings

Stefan Peeters[1*], Takuya Kuwahara[1,2], Fabian Härtwig[3,4], Stefan Makowski[3], Volker Weihnacht[3], Andrés Fabián Lasagni[3,4], Martin Dienwiebel[1,5], Michael Moseler[1,6], Gianpietro Moras[1]

[1]*Fraunhofer IWM, MikroTribologie Centrum µTC, Wöhlerstraße 11, 79108 Freiburg, Germany*

[2]*Osaka Metropolitan University, 3-3-138 Sugimoto, Sumiyoshi-ku, 558-8585 Osaka, Japan*

[3]*Fraunhofer IWS, Winterbergstraße 28, 01277 Dresden, Germany*

[4]*Technische Universität Dresden, Institut für Fertigungstechnik, George-Bähr-Straße 3c, 01069 Dresden*

[5]*Karlsruhe Institute of Technology (KIT), IAM – Institute for Applied Materials, MikroTribologie Centrum µTC, Straße am Forum 7, 76131 Karlsruhe, Germany*

[6]*University of Freiburg, Institute of Physics, Herrmann-Herder-Straße 3, 79104 Freiburg, Germany*

*Corresponding author: stefan.peeters@iwm.fraunhofer.de

S1 – Surface roughness of the lapped coatings measured by white light interferometry

The roughness of the coatings was measured after lapping using 3D white light interferometry (Keyence VK-X3000, Osaka, Japan). Using a 50x objective, the resulting image sizes were 289.5 × 217.1 μm^2 (2048 pixel × 1536 pixel). The images were taken at multiple positions, and the results were averaged. The roughness parameters were calculated using an S-Filter of 0.5 µm and an L-Filter of 0.08 mm.

Table S1 reports the arithmetical mean height *Sa*, the maximum height *Sz* and the root mean square height Sq. The arithmetical mean height is calculated as $Sa = \frac{1}{A}\iint |h(x,y)|\, dx\, dy$, where $h(x,y)$ is the height map and A is its area. The maximum height *Sz* is the sum of the largest peak height value and the largest pit depth value of the height map. The root mean square height *Sq* is defined in Equation 1 in the main text, where it is introduced as h'_{RMS}. The small differences in the roughness of the coatings show no correlation with the friction coefficient.

Table S1. Surface roughness parameters measured by white light interferometry.

Coating	Sa (µm)	Sz (µm)	Sq (µm)
**ta-C**	0.0084 ± 0.0001	1.5414 ± 0.2352	0.0356 ± 0.0062
**a-C**	0.0146 ± 0.0021	3.4700 ± 0.9977	0.0548 ± 0.0184
**ta-C:B**	0.0038 ± 0.0006	1.1618 ± 0.0511	0.0083 ± 0.0001
**a-C:B**	0.0070 ± 0.0003	1.1601 ± 0.2665	0.0128 ± 0.0030

S2 – Details on wear measured by profilometry

Table S2. Results of the profilometry measurements of the wear scars for each tribological test.

Coating	Max. depth (µm)	Area (µm^2)
**ta-C**	0.4	51.36
	0.0	1.65
	0.0	3.27
**a-C**	0.1	6.87
	0.1	7.65
	0.1	7.56
**ta-C:B**	1.0	160.28
	0.8	140.07
	0.8	134.20
**a-C:B**	0.1	10.07
	0.1	10.58
	0.1	9.66

S3 – Topographies of the wear scars by atomic force microscopy

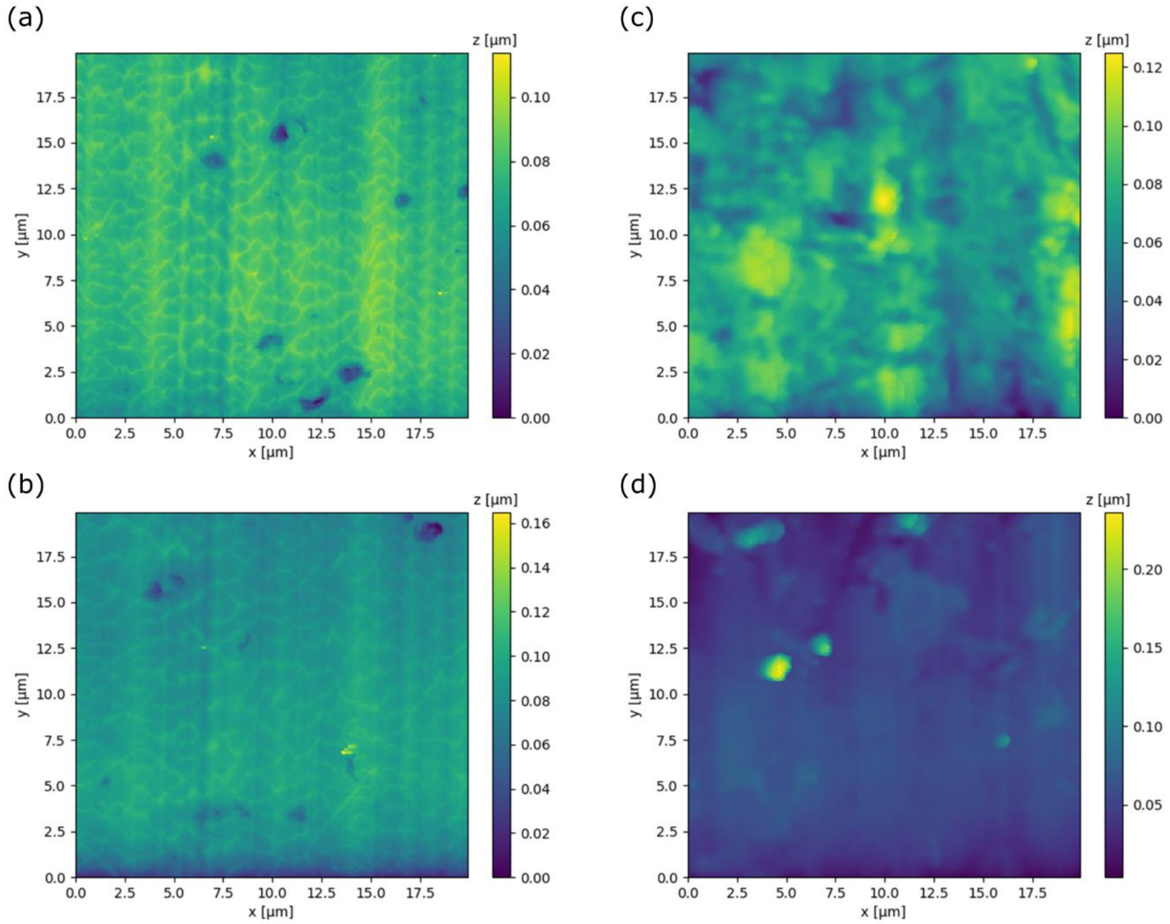

Figure S1. AFM topographies of the wear scars on a-C (a, b) and the corresponding topographies on the Al_2O_3 ball (c, d).

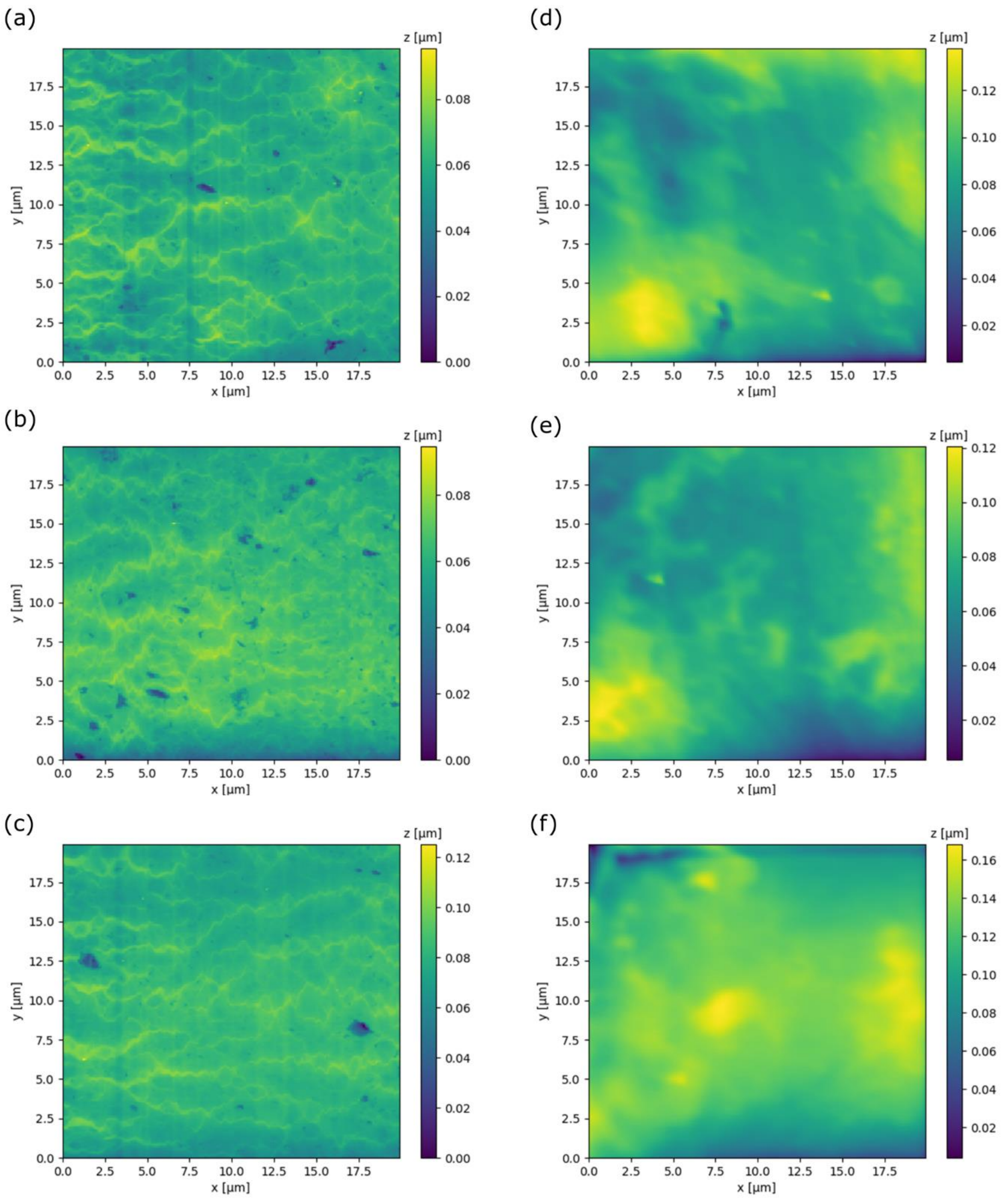

Figure S2. AFM topographies of the wear scars on ta-C (a-c) and the corresponding topographies on the Al_2O_3 ball (d-f).

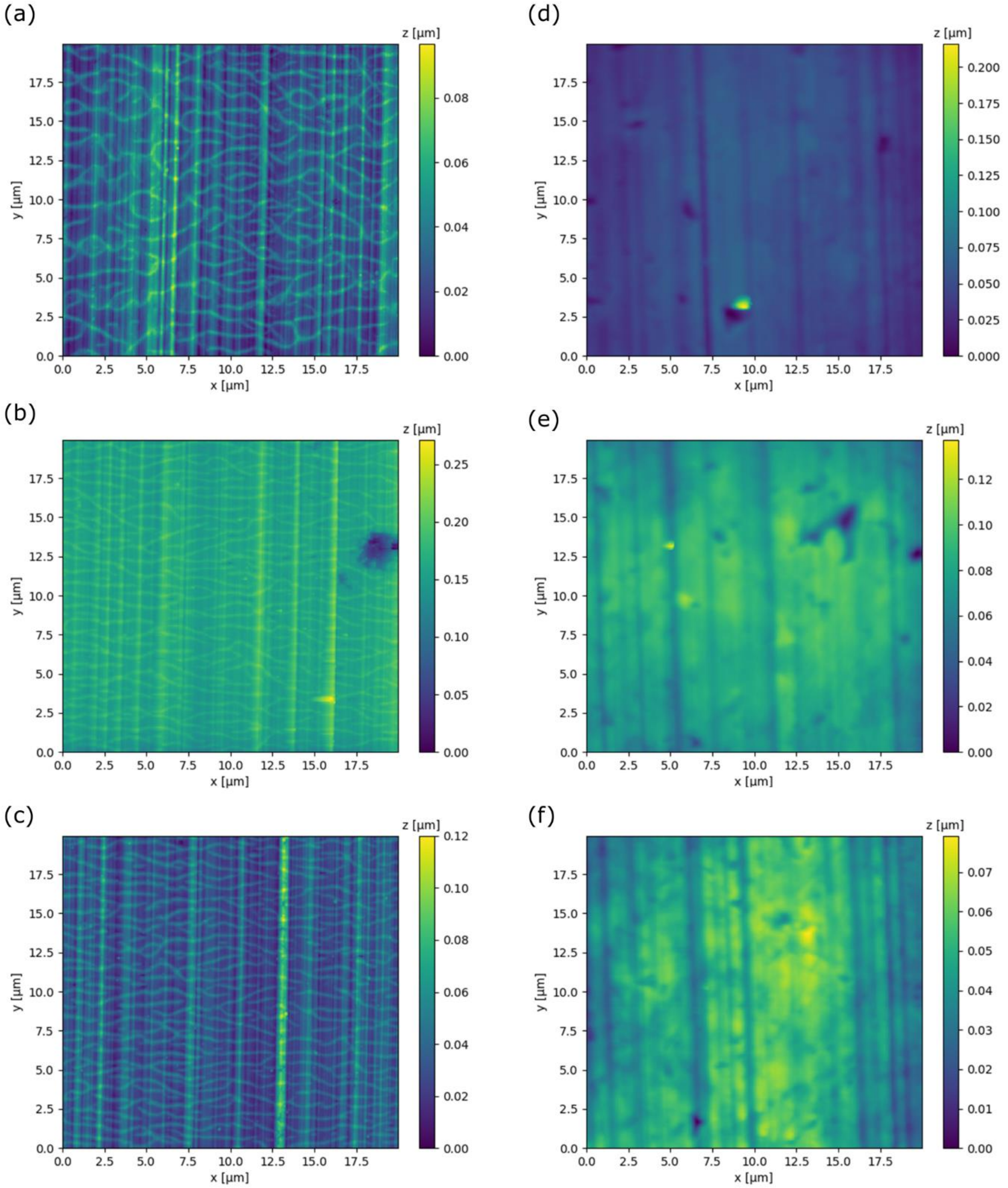

Figure S3. AFM topographies of the wear scars on a-C:B (a-c) and the corresponding topographies on the Al_2O_3 ball (d-f).

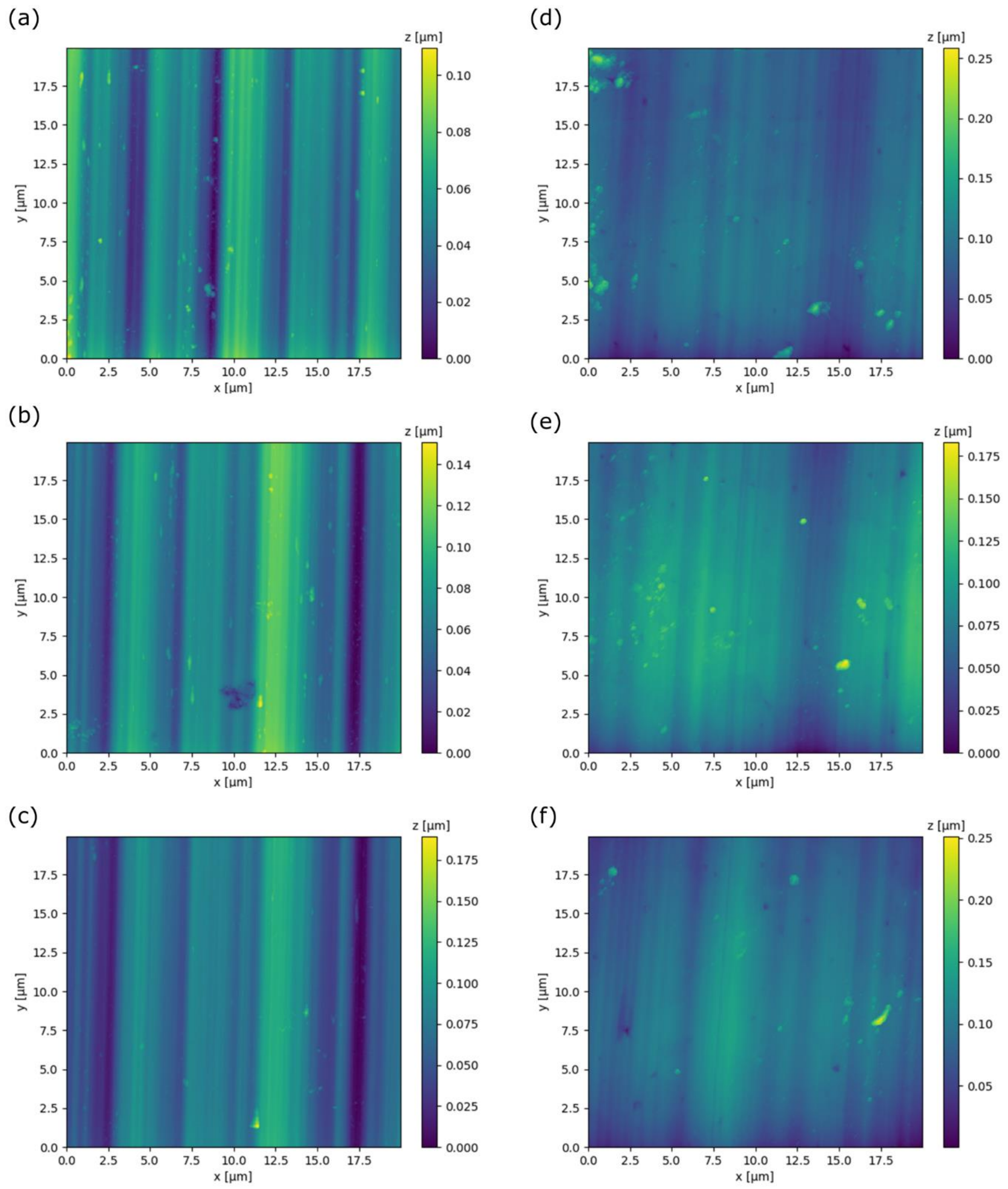

Figure S4. AFM topographies of the wear scars on ta-C:B (a-c) and the corresponding topographies on the Al_2O_3 ball (d-f).

S4 – XPS analysis of the undoped and B-doped films

The XPS spectra of the wear scar and of the pristine surface of ta-C:B were taken with a PHI 5000 VersaProbe II system (ULVAC-PHI, Chigasaki, Japan). The system uses a monochromatic, microfocused Al Kα x-ray source with an excitation energy of 1486.6 eV. A spot size of $100 \times 100\ \mu m^2$ was used. The XPS measurements were performed with a dual-beam neutralizer for charge compensation.

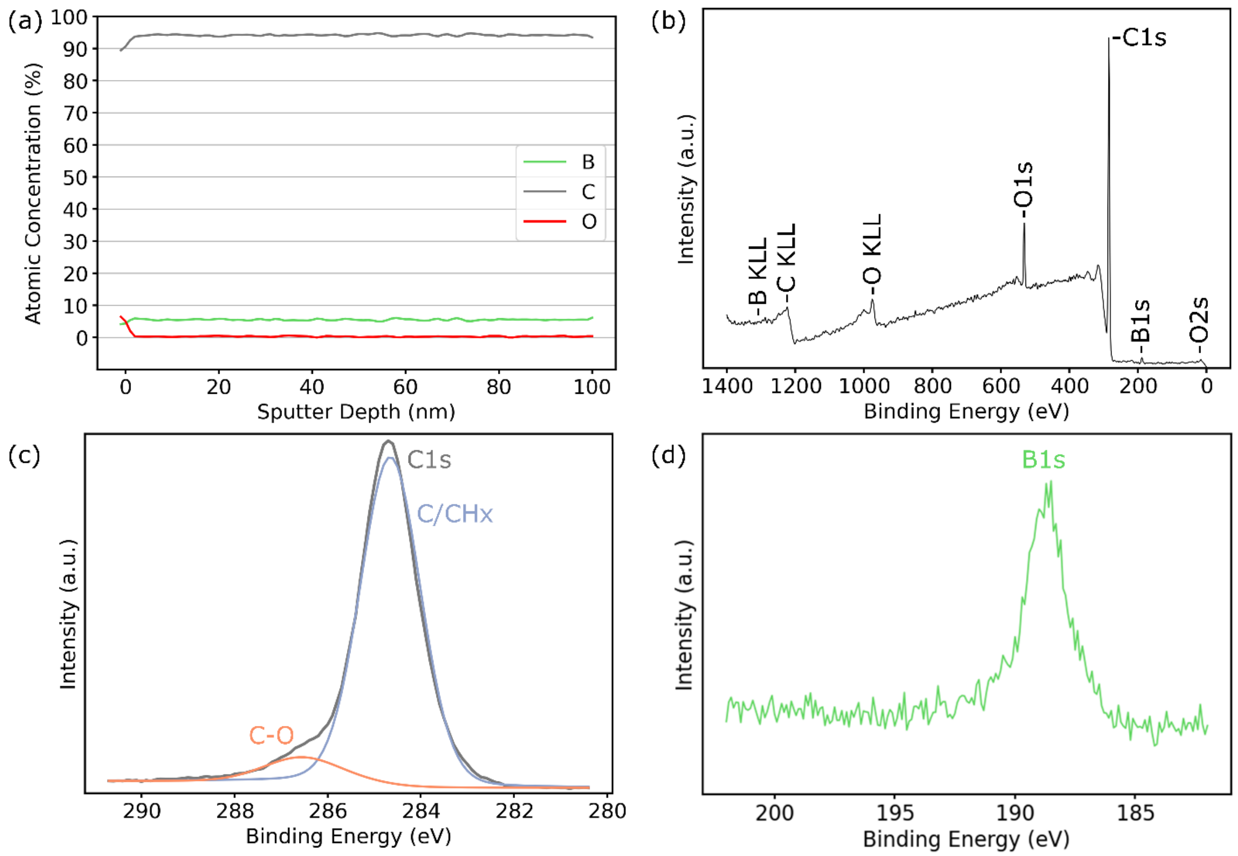

Figure S5. XPS analysis of the wear track of ta-C:B. (a) Depth profile. The B concentration on the surface is slightly lower (3.8 at.%) than in the bulk material (~5 at.%). The surface is slightly oxidized (7.3 at.%). (b) Full XPS spectrum. (c) C1s peak (with the C-O peak accounting for 7.5% of the total peak area. (d) B1s peak.

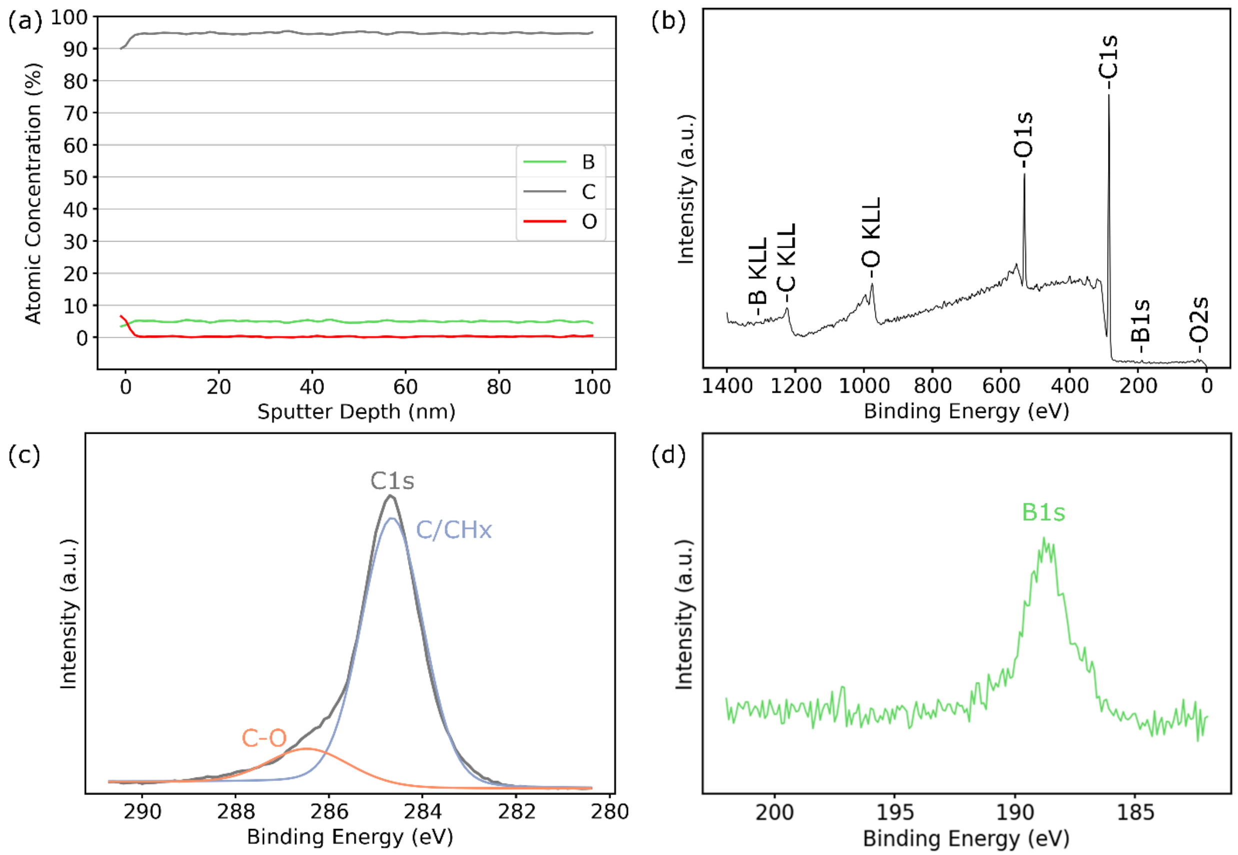

Figure S6. XPS analysis of the pristine surface of ta-C:B. (a) Depth profile. The B concentration on the surface is slightly lower (3.3 at.%) than in the bulk material (~5 at.%). The surface is slightly oxidized (11.7 at.%). (b) Full XPS spectrum. (c) C1s peak (with the C-O peak accounting for 13.7% of the total peak area. (d) B1s peak.

S5 – Raman spectra of the coatings

Raman spectra were recorded by an "inVia" Raman microscope from Renishaw plc., UK (50x objective, wavelength 514 nm, laser power 8 mW, CCD line detector). Each sample was measured at three different locations on the coating's surface, and only a single representative measurement out of three is shown in Figure S1 for each coating. While the spectra of ta-C, ta-C:B and a-C:B were recorded on the same samples used in the tribological experiments, the spectrum of a-C was obtained on a different sample. Nevertheless, the deposition parameters of the two a-C samples were almost identical, therefore their structure and their spectra should be equivalent.

Figure S7 shows that coatings with similar hardness are also structurally similar, regardless of the presence of boron. Both spectra of a-C and a-C:B are characterized by the presence of a D shoulder, indicating a mild structuring of the sp^2 phase, as expected by these softer materials. This shoulder cannot be clearly observed in the case of the harder ta-C and ta-C:B coatings, which are mainly composed of sp^3 carbon.

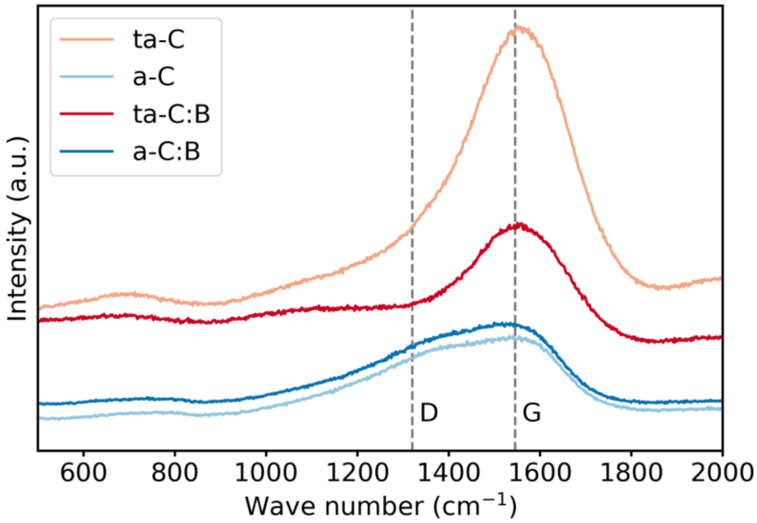

Figure S7. Raman spectra of the pristine surface of the coatings.
